# Supplementary material for: Complexome profiling of the Chlamydomonas psb28 mutant reveals TEF5 as an early PSII assembly factor
Source: Plant Cell. 2025 Mar 17;37(6):koaf055. doi: 10.1093/plcell/koaf055 (PMC12164590; doi:10.1093/plcell/koaf055)
Supplement: koaf055_Supplementary_Data [file koaf055_supplementary_data.zip › psb28_interactive.zip!/plots/intensity/qualityPlots/peptides/CLPP4_Cre12.g500950.t1.2.html]

### Cre12.g500950.t1.2

Trivial name: CLPP4  
SeqCoverage [median]: 4.30  
Mass: 38.41 kDa  
  
 

| Sequence | MissedCleavage | Mass [Da] | wtIntensity | muIntensity | Score |
| --- | --- | --- | --- | --- | --- |
| IKEEFVTDKDDMVKR | 3 | 1851.94 | 612.70 | 2404.57 | 79.77 |
|  |  |  | 612.7 | 2404.6 |  |
